# Two draft genome sequences of *Pseudomonas jessenii* strains isolated from a copper contaminated site in Denmark

**DOI:** 10.1186/s40793-016-0200-8

**Published:** 2016-11-03

**Authors:** Yanan Qin, Dan Wang, Kristian K. Brandt, Christopher Rensing

**Affiliations:** 1 Department of Plant and Environmental Sciences, University of Copenhagen, Frederiksberg, Denmark; 2 State Key Laboratory of Agricultural Microbiology, College of Life Sciences and Technology, Huazhong Agricultural University, Wuhan, China; 3 College of Resources and the Environment, Fujian Agriculture and Forestry University, Fuzhou, China; 4 J. Craig Venter Institute, La Jolla, CA USA

**Keywords:** *Pseudomonas jessenii*, Comparative genomics, Copper resistance

## Abstract

*Pseudomonas jessenii* C2 and *Pseudomonas jessenii* H16 were isolated from low-Cu and high-Cu industrially contaminated soil, respectively. *P. jessenii* H16 displayed significant resistance to copper when compared to *P. jessenii* C2. Here we describe genome sequences and interesting features of these two strains. The genome of *P. jessenii* C2 comprised 6,420,113 bp, with 5814 protein-coding genes and 67 RNA genes. *P. jessenii* H16 comprised 6,807,788 bp, with 5995 protein-coding genes and 70 RNA genes. Of special interest was a specific adaptation to this harsh copper-contaminated environment as *P. jessenii* H16 contained a novel putative copper resistance genomic island (GI) of around 50,000 bp.

**Electronic supplementary material:**

The online version of this article (doi:10.1186/s40793-016-0200-8) contains supplementary material, which is available to authorized users.

## Introduction

Copper is an essential micronutrient in most organisms and required as a co-factor in biological processes such as redox reactions (electron transport, oxidative respiration, denitrification) [1, 2]. However, at higher concentrations copper will become toxic and inhibit or kill cells. Therefore, microorganisms have developed sophisticated copper homeostasis and resistance mechanisms in order to maintain the normal cellular copper supply to essential cuproenzymes while avoiding copper poisoning [3, 4]. Some highly copper resistant microorganisms have attracted great interests due to potential biotechnological applications in bio-mining and bioremediation of environments contaminated with copper [5].

*Pseudomonas* spp. are ubiquitous inhabitants of soil, water and plant surfaces belonging to the *Gammaproteobacteria*. *Pseudomonas* spp. has an exceptional capacity to produce a wide variety of secondary metabolites, including antibiotics that are toxic to plant pathogens [6, 7]. *Pseudomonas jessenii* was also found to be an important rhizobacterium conferring protection against a number of soilborne plant pathogens [8]. *P. jessenii* C2 and *P. jessenii* H16 were isolated from low-Cu soil and high-Cu soil from an abandoned wood impregnation site in Hygum, Denmark, respectively [9]. The Hygum site was contaminated with copper sulfate from 1911 to 1924, then the area was cultivated until 1993 and has been a fallow field since then [9, 10]. *P. jessenii* H16 was able to grow in medium containing high concentrations of copper, whereas *P. jessenii* C2 was sensitive to high copper concentrations. Here, we present the genome sequences, a brief characterization and annotation of *P. jessenii* C2 and *P. jessenii* H16.

## Organism information

### Classification and features

A highly copper contaminated high-Cu soil and a corresponding low-Cu soil were collected (0–20 cm depth) from a well-described Cu gradient field site in Hygum, Denmark. The high-Cu site was contaminated almost exclusively with CuSO_4_ more than 90 years ago [9]. The adjacent low-Cu control site was located just outside the contaminated area and had been subjected to the same land use for more than 80 years. The low-Cu and high-Cu soil had similar physicochemical characteristics except for their total Cu contents of 21 and 3172 mg kg^-1^, respectively [9, 11]. Bacteria were isolated from replicated soil subsamples (*n* = 3) and diluted, spread-plated on *Pseudomonas* *-*selective Gould’s S1 agar [11]. For each dilution series, 30 colonies emerging after two days at 25 °C were selected and isolated in pure culture by repeated plating [11]. Two of the resulting isolates were selected for further study. *P. jessenii* H16 was able to grow at high concentration of Cu (2 mM) on one-tenth strength LB agar, whereas *P. jessenii* C2 only grew with up to 0.125 mM Cu.

**Fig. 1:**
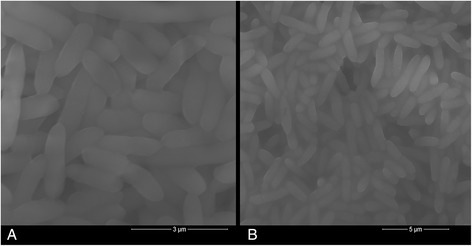
Micrograph of *Pseudomonas jessenii* C2 and H16 obtained by scanning electron microscopy. **a** *Pseudomonas jessenii* C2. **b** *Pseudomonas jessenii* H16

Strain C2 and H16 were both Gram-reaction negative. Cells of strain C2 and H16 were rod shaped with rounded ends and motile. The cells of C2 were 2.12–2.45 μm (mean, 2.28 μm) in length compared to 0.49–0.62 μm (mean, 0.55 μm) in width (Fig. 1a). The cells of H16 were 1.95–2.38 μm × 0.42–0.57 μm in size (Fig. 1b). No Sporulation was observed for both strains. The colonies were white and translucent on Gould’s S1 agar medium. Growth occurred aerobically at 4–37 °C, and optimal growth was observed at 30 °C, pH 7.0 for strain C2. Strain H16 preferred pH 6.7, at 30 °C for optimal growth. Both strains grew in 0–4 % (w/v) NaCl (Tables 1 and 2).

**Table 1:** Classification and general features of *P.jessenii* C2 according to the MIGS recommendations [15]

MIGS ID	Property	Term	Evidence code^a^
	Classification	Domain *Bacteria*	TAS [40]
		Phylum *Proteobacteria*	TAS [41]
		Class *Gammaproteobacteria*	TAS [42, 43]
		Order *Pseudomonadales*	TAS [44]
		Family *Pseudomonadaceae*	TAS [45]
		Genus *Pseudomonas*	TAS [46, 47]
		Species *P. jessenii*	TAS [48]
		strain: *C2*	IDA
	Gram stain	Negative	IDA
	Cell shape	Rod-shaped	IDA
	Motility	Motile	IDA
	Sporulation	Non-sporulating	IDA
	Temperature range	4–37 °C	IDA
	Optimum temperature	30 °C	IDA
	Optimum pH	7.0	IDA
	Carbon source	d-glucose, d-melibiose, d-sucrose, d-mannitol, L-rhamnose, inositol, trehalose, d-lyxose,L-arabinose	IDA
MIGS-6	Habitat	soil	IDA
MIGS-6.3	Salinity	0–4 %	IDA
MIGS-22	Oxygen requirement	Aerobic	IDA
MIGS-15	Biotic relationship	Free-living	IDA
MIGS-14	Pathogenicity	Non-pathogen	NAS
MIGS-4	Geographic location	Hygum, Denmark	IDA
MIGS-5	Sample collection	May 2013	IDA
MIGS-4.1	Latitude	55° 46’ 25’’N	IDA
MIGS-4.2	Longitude	9° 25’ 52’’ E	IDA

^a^Evidence codes - *IDA* inferred from direct assay, *TAS* traceable author statement (i.e., a direct report exists in the literature), *NAS* non-traceable author statement (i.e., not directly observed for the living, isolated sample, but based on a generally accepted property for the species, or anecdotal evidence). These evidence codes are from the Gene Ontology project [49]. If the evidence is IDA, the property was directly observed by the authors

**Table 2:** Classification and general features of *P.jessenii* H16 according to the MIGS recommendations [15]

MIGS ID	Property	Term	Evidence code^a^
	Classification	Domain *Bacteria*	TAS [40]
		Phylum *Proteobacteria*	TAS [41]
		Class *Gammaproteobacteria*	TAS [42, 43]
		Order *Pseudomonadales*	TAS [44]
		Family *Pseudomonadaceae*	TAS [45]
		Genus *Pseudomonas*	TAS [46, 47]
		Species *P. jessenii*	TAS [48]
		strain: *H16*	IDA
	Gram stain	Negative	IDA
	Cell shape	Rod-shaped	IDA
	Motility	Motile	IDA
	Sporulation	Non-sporulating	IDA
	Temperature range	4–37 °C	IDA
	Optimum temperature	30 °C	IDA
	Optimum pH	6.7	IDA
	Carbon source	d-glucose, d-melibiose, d-sucrose, d-mannitol, trehalose, d-lyxose, L-arabinose,inostitol	IDA
MIGS-6	Habitat	Copper contaminated soil	IDA
MIGS-6.3	Salinity	0–4 %	IDA
MIGS-22	Oxygen requirement	Aerobic	IDA
MIGS-15	Biotic relationship	Free-living	IDA
MIGS-14	Pathogenicity	Non-pathogen	NAS
MIGS-4	Geographic location	Hygum, Denmark	IDA
MIGS-5	Sample collection	May 2013	IDA
MIGS-4.1	Latitude	55° 46’ 25’’N	IDA
MIGS-4.2	Longitude	9° 25’ 52’’ E	IDA

^a^Evidence codes - *IDA* inferred from direct assay, *TAS* traceable author statement (i.e., a direct report exists in the literature), *NAS* non-traceable author statement (i.e., not directly observed for the living, isolated sample, but based on a generally accepted property for the species, or anecdotal evidence). These evidence codes are from the Gene Ontology project [49]. If the evidence is IDA, the property was directly observed by the authors

#### Chemotaxonomy

Fatty acid analyses were performed by the Identification Service of the DSMZ, Braunschweig, Germany [12]. The fatty acid profiles were similar when comparing strains C2 and H16. The major fatty acids of the two strains showed as follows: C_16: 1_ *ω*7*c* and/or iso-C_15: 0_ 2-OH (36.4 % in *P. jessenii* C2 and 40.1 % in *P. jessenii* H16); C_18 : 1_ *ω*7*c* (15.3 % in *P. jessenii* C2 and 10.8 % in *P. jessenii* H16) and C_16 : 0_ (28.8 % in *P. jessenii* C2 and 34.6 % *P. jessenii* H16).

Biochemical properties were tested using API 20NE (BioMérieux) for Strains C2 and H16. In the API 20NE system, positive reactions for both strains were observed for nitrate reduction and production of arginine dihydrolase; negative reactions were observed for indole production, urease activity, Lysine and ornithine decarboxylase and gelatin hydrolysis (Additional file 1: Table S1). Strain C2 assimilated d-glucose, d-melibiose, d-sucrose, d-mannitol, l-rhamnose, inositol, trehalose, d-lyxose and l-arabinose, but not sorbitol. Strain H16 could utilize d-glucose, d-melibiose, d-sucrose, d-mannitol, trehalose, d-lyxose, l-arabinose and inostitol as carbon sources, but not, l-rhamnose and sorbitol (Additional file 1: Table S1).

#### 16S rRNA gene analysis

**Fig. 2:**
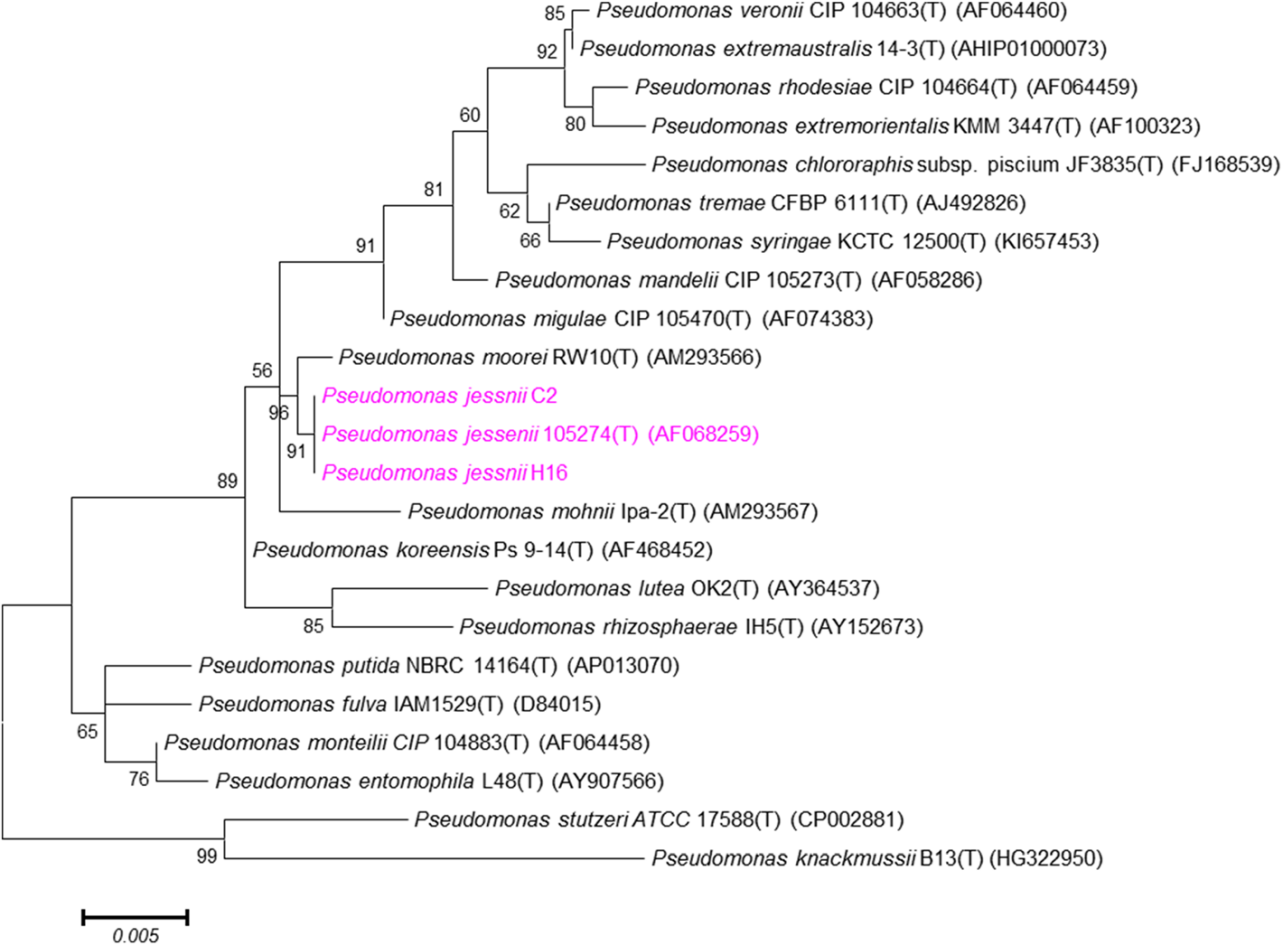
Phylogenetic tree of *P. jessenii* C2 and *P. jessenii* H16 relative to type strains within the genus *Pseudomonas*. The strains and their corresponding GenBank accession numbers of 16S rRNA genes are displayed in parentheses. The sequences were aligned using Clustal W, and the maximum likelihood tree was constructed based on Jukes-Cantor model by using MEGA6 [14]. Bootstrap values above 50 % are shown obtained from 1000 bootstrap replications. Bar 0.005 substitutions per nucleotide position

Comparative 16S rRNA gene sequence analysis using the EzTaxon database [13] indicated that strain C2 and H16 were both most closely related to *P. jessenii* CIP 105275^T^ (GenBank accession no. AF068259) with sequence similarities of 99.87 and 99.14 %, respectively. Phylogenetic analysis was performed using the 16S rRNA gene sequences of strains C2, H16 and related species. Sequences were aligned and phylogenic trees were constructed using Maximum Likelihood method implemented in MEGA version 6 [14]. The resultant tree topologies were evaluated by bootstrap analyses with 1000 random samplings (Fig. 2).

## Genome sequencing information

### Genome project history

Next-generation shotgun-sequencing was performed at the Beijing Genomics Institute (BGI, Shenzhen). The whole genome shotgun project of *P. jessenii* C2 and *P. jessenii* H16 has been deposited at DDBJ/EMBL/GenBank under the accession numbers JSAK00000000 and JSAL00000000. The version described in this paper is the first version. A summary of the project and the Minimum Information about a Genome Sequence [15] are shown in Table 3.

**Table 3:** Project information

MIGS ID	Property	Term	
MIGS 31	Finishing quality	High-quality draft	High-quality draft
MIGS-28	Libraries used	One paired-end Illumina library	One paired-end Illumina library
MIGS 29	Sequencing platforms	llIumina HiSeq 2000	llIumina HiSeq 2000
MIGS 31.2	Fold coverage	170×	160×
MIGS 30	Assemblers	CLC Genomics Workbench, version7.0.4	CLC Genomics Workbench, version7.0.4
MIGS 32	Gene calling method	Glimmer 3.0	Glimmer 3.0
	Locus Tag	NL64	RY26
	Genbank ID	JSAK00000000.1	JSAL00000000.1
	GenBank Date of Release	2014/12/17	2014/12/17
	GOLD ID	Gp0157184	Gp0157185
	BIOPROJECT	PRJNA264019	PRJNA264019
MIGS 13	Source Material Identifier	HC-Cu02	HC_Cu16
	Project relevance	Low-Cu soil	Copper contaminated soil

### Growth conditions and genomic DNA preparation

*P. jessenii* C2 and *P. jessenii* H16 were aerobically cultivated on *Pseudomonas*-selective Gould’s S1 agar at 28 °C [16]. Total genomic DNA was extracted using Puregene Yeast/Bact Kit according to the manufacturer’s instructions (QIAGEN). The quantity of the genomic DNA was determined by Qubit® fluorometer (Invitrogen, CA, USA) with Qubit dsDNA BR Assay kit (Invitrogen, CA, USA) and amounted to 55 ng/μL of DNA for *P. jessenii* C2 and 48.2 ng/μL of DNA for *P. jessenii* H16.

### Genome sequencing and assembly

The genome sequence of *P. jessenii* H16 and *P. jessenii* C2 was determined by BGI using the Illumina Hiseq2000 with a 500 bp library constructed [17], generating 1.09 gigabytes of DNA sequence with an average coverage of ~160 fold and ~170 fold; yielding 1,205,9244 and 1,203,8756 paired-end reads with a 90-bp read length, respectively. The resulting sequence data was quality assessed, trimmed, and assembled *de novo* as described previously [18] using CLCBio Genomic Workbench 7.0 (CLCBio, Denmark). *P. jessenii* H16 generated 78 contigs with an n50 value of 279,014 bp. *P. jessenii* C2 generated 64 contigs with an n50 value of 224,893 bp.

### Genome annotation

The genes in the assembled genome were predicted based on the RAST database [19]. The predicted ORFs were annotated by searching clusters of orthologous groups [20] using the SEED database [21]. RNAmmer 1.2 [22] and tRNAscanSE 1.23 [23] were used to identify rRNA and tRNA genes, respectively.

## Genome properties

**Fig. 3:**
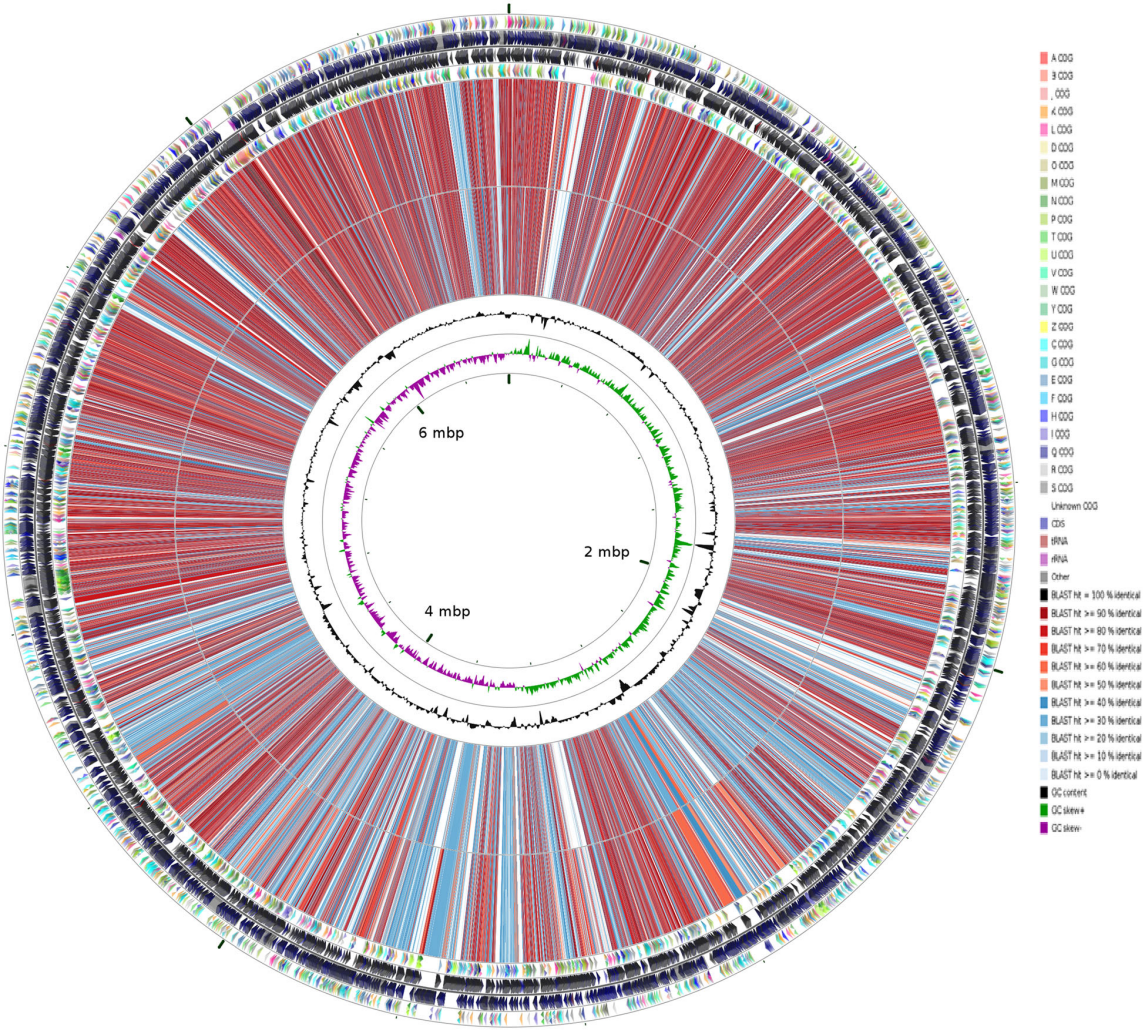
Circular map of the chromosome of *P. jessenii* C2 and *P. jessenii* H16. From outside to the center: *P. jessenii H16* genes on forward strand (color by COG categories), *P. jessenii H16* CDS on forward strand, tRNA, rRNA, other; *P. jessenii* H16 CDS on reverse strand, *P. jessenii* H16 tRNA, rRNA, other, genes on reverse strand (color by COG categories); *P. jessenii* C2 CDS blast with *P. jessenii* H16 CDS; *P. fluorescens* SW25 (NC_012660) CDS blast with *P. jessenii* H16 CDS; *P. jessenii* H16 GC content; *P. jessenii* H16 GC skew, where *green* indicates positive values and magenta indicates negative values

*P. jessenii* C2 contained 6,420,113 bp with a G+C content of 59.83 %, 5881 predicted genes, 5814 were protein-coding genes, 63 tRNA genes and 4 rRNA genes. In total, 5179 genes were assigned to biological functions and 635 were annotated as hypothetical proteins. *P. jessenii* H16 contained 6,807,788 bp, with a GC content of 59.02 %, 6065 predicted genes, and 5995 were protein-coding genes, 65 tRNA and 5 rRNA genes. Among the protein coding genes 5344 were assigned to biological functions, while 651 were annotated as hypothetical proteins. The properties and statistics of those two genomes are summarized in Table 4. The distribution of genes into COG functional categories is presented in Table 5 and Fig. 3.

**Table 4:** Genome statistics

Attribute	*P. jessenii* C2		*P. jessenii* H16	
	Value	% of total	Value	% of total
Genome size (bp)	6,420,113	100.00	6,807,788	100.00
DNA coding (bp)	5,484,120	85.42	5,835,906	85.72
DNA G+C (bp)	3,851,154	59.83	4,017,956	59.02
DNA scaffolds	64	-	78	-
Total genes	5881	100.00	6065	100.00
Protein coding genes	5814	98.86	5995	98.85
RNA genes	67	1.14	70	1.15
Pseudo genes				
Genes with function prediction	5179	88.06	5344	88.11
Genes assigned to COGs	4314	73.75	4354	71.79
Genes with Pfam domains	3595	61.13	3587	59.14
Genes with signal peptides	510	8.67	537	8.85
Genes with transmembrane helices	1260	21.42	1343	22.14
CRISPR repeats	38	-	11	-

**Table 5:** Number of genes associated with general COG functional categories

	*P. jessenii* C2		*P. jessenii* H16		
Code	Value	%^a^	Value	%^a^	Description
J	183	3.14	186	3.10	Translation, ribosomal structure and biogenesis
A	1	0.02	2	0.03	RNA processing and modification
K	425	7.31	425	7.09	Transcription
L	147	2.53	135	2.25	Replication, recombination and repair
B	2	0.34	3	0.05	Chromatin structure and dynamics
D	35	0.60	35	0.58	Cell cycle control, Cell division, chromosome partitioning
V	59	1.01	57	0.95	Defense mechanisms
T	368	6.33	389	6.49	Signal transduction mechanisms
M	239	4.11	282	4.70	Cell wall/membrane biogenesis
N	128	2.20	135	2.25	Cell motility
U	119	2.05	128	2.14	Intracellular trafficking and secretion
O	175	3.01	168	2.80	Posttranslational modification, protein turnover, chaperones
C	312	5.37	278	4.64	Energy production and conversion
G	219	3.77	247	4.12	Carbohydrate transport and metabolism
E	515	8.86	497	8.29	Amino acid transport and metabolism
F	85	1.46	99	1.65	Nucleotide transport and metabolism
H	177	3.04	193	3.22	Coenzyme transport and metabolism
I	237	4.08	194	3.24	Lipid transport and metabolism
P	300	5.16	286	4.77	Inorganic ion transport and metabolism
Q	142	2.44	129	2.15	Secondary metabolites biosynthesis, transport and catabolism
R	532	9.15	572	9.54	General function prediction only
S	444	7.64	451	7.52	Function unknown
-	970	16.68	1104	18.42	Not in COGs

^a^The total is based on the total number of protein coding genes in the genome

## Insights into the genome

Genes conferring resistances to heavy metals in the two studied strains are listed in Table 6. Copper efflux from the cytosol is mediated by the P_1B_-type ATPase family, which is highly conserved from bacteria to humans [24]. Both *P. jessenii* C2 and *P. jessenii* H16 contained genes encoding a copper-transporting P_1B_-type ATPase (CopA) with conserved CPCALG motif [25], a copper-responsive metalloregulatory protein CueR, and the multicopper oxidase CueO. In addition, one additional gene encoding a Cu^+^-ATPase is present on the genome of *P. jessenii* H16 as part of the GI discussed later. *P. jessenii* H16 also contained *ccoI* encoding a Cu^+^-ATPase catalyzing a slower rate of efflux for copper insertion into cytochrome c oxidase [26]. The presence of a *cop* operon, comprising *copABCDRS* had been reported in related *P.fluorescens* SBW25 and *P.putida* KT2440 [27, 28]. Both *P. jessenii* strains contained *copCDRS* probably encoding proteins responsible for copper uptake, however, only *P. jessenii* H16 also contained *copAB* as part of the GI. Both *P. jessenii* C2 and *P. jessenii* H16 contain an arsenic resistance determinant (*arsRBCH*) [29] a gene involved in chromate resistance (*chrA*) [26] (Table 6). The two strains also contained genes encoding a multidrug efflux system MexEF-OprN regulated by MexT and genes encoding DNA gyrase subunit A and B, and topoisomerase subunit (IV) A and B [30, 31].

**Table 6:** *P.jessenii* C2 and *P.jessenii* H16 genes related to heavy metal resistance

*P.jessenii* C2		*P.jessenii* H16		
Protein id	Size/aa	Protein id	Size/aa	Predicted function
KII28258	513	KII28679	459	Multicopper oxidase CueO-1
KII31612	122	KII28987	121	Copper resistance protein CopC
KII31613	282	KII28988	286	Copper resistance protein CopD-1
KII30013	133	KII32596	138	Cu(I)-responsive transcriptional regulator CopR
KII30014	798	KII32595	798	Copper-translocating P-type ATPase CopA-1
KII30016	66	KII32593	66	Copper resistance protein CopZ
KII37329	149	KII29565	149	Metal-binding protein CopG-1
KII33434	179	KII28041	179	Copper tolerance protein
KII33435	227	KII28042	227	Copper response regulator CusR-1
KII33436	450	KII28043	450	Copper sensor histidine kinase CusS-1
KII34384	759	KII35062	770	Lead, cadmium, zinc and mercury transporting ATPase
KII29503	231	KII36596	231	Arsenic resistance protein ArsH
KII29504	157	KII36597	157	Arsenate reductase ArsC
KII29505	428	KII36598	116	Arsenical resistance operon repressor ArsR
KII29506	116	KII36460	428	Arsenical pump membrane protein ArsB
KII31669	453	KII30277	447	Chromate transport protein ChrA
		KII37024	798	Cytochrome c oxidases
		KII37706	1047	Cation transporter CusA
		KII37707	494	RND transporter CusB
		KII37708	418	RND efflux outer membrane protein CusC
		KII37709	312	Copper resistance protein CopD-2
		KII37710	462	Copper sensor histidine kinase CusS-2
		KII37711	231	Copper response regulator CusR-2
		KII37713	178	Blue (type1) copper domain-containing protein
		KII37893	676	Copper-translocating P-type ATPase CopA-2
		KII37715	642	Multicopper oxidase CueO-2
		KII37716	333	Copper resistance protein CopB
		KII37717	155	Metal-binding protein CopG-2
		KII37719	321	Cation transporter CzcD
		KII37721	436	Nickel efflux system NrcA
		KII37723	99	Nickel resistance protein NcrB
		KII37733	116	Mercuric transport protein MerT
		KII37734	91	Mercury transporter MerR
		KII37735	144	Mercury transport protein MerC
		KII37736	560	Mercuric reductase MerA
		KII37737	212	Alkylmercury lyase MerB

**Fig. 4:**
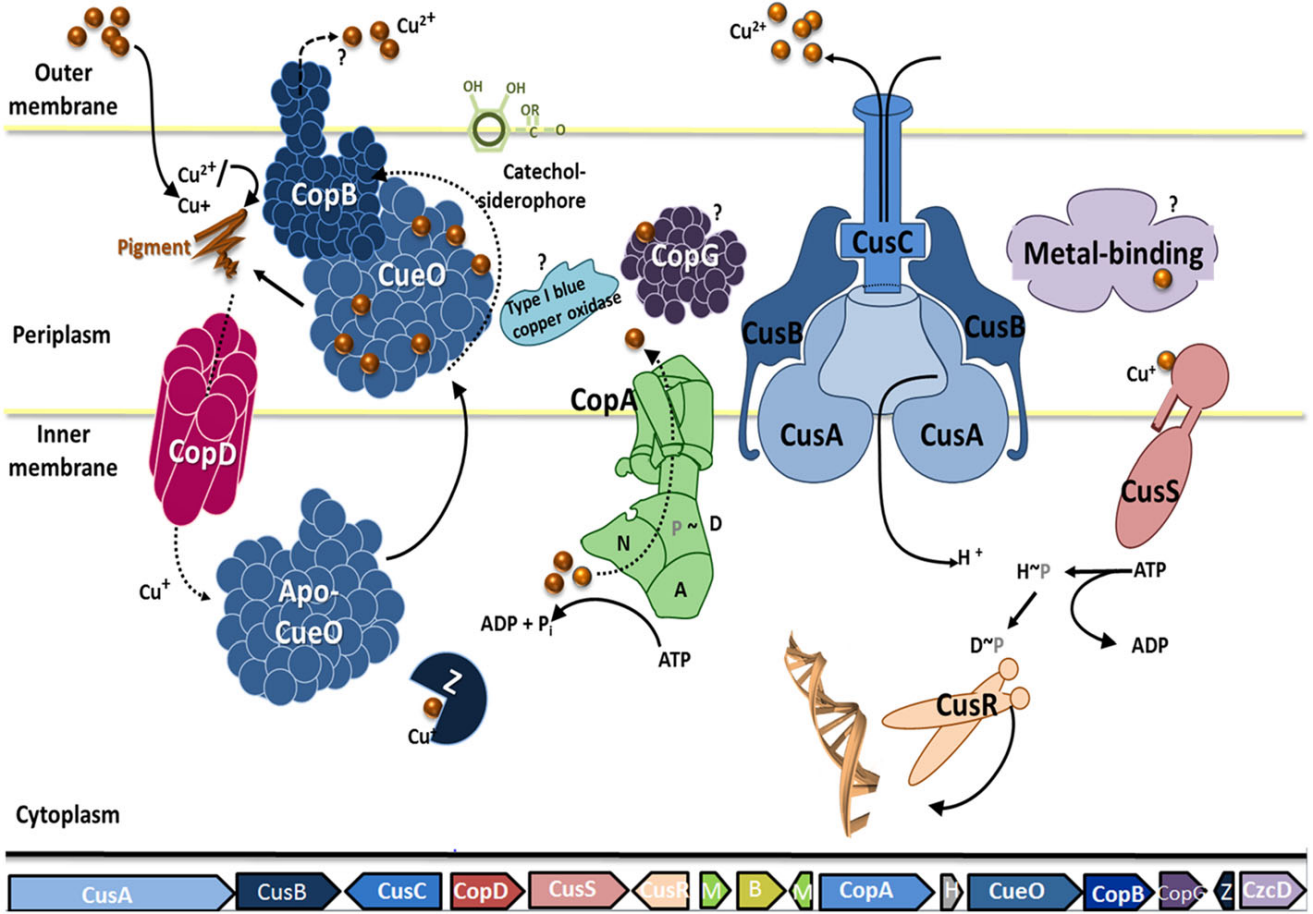
Putative copper fitness/pathogenicity island in *P.jessenii* H16. Model of encoded proteins involved in copper resistance. *CusA* copper transporter, *CusB* RND transporter, *CusC* RND efflux outer membrane protein, *CopD* copper resistance protein, *CusS-2* copper sensor histidine kinase, *CusR-2* copper response regulator, *CopA-2* copper-translocating P-type ATPase, *CueO-2* multicopper oxidase, *CopB* copper resistance protei, *CopG-2* metal-binding protein, *CzcD* cation transporter, *B* blue (type1) copper domain-containing protein CinA, *H* hypothetical protein, *M* putative metal-binding protein, *Z* copper chaperone

*P. jessenii* H16 contained an additional putative metal fitness/pathogenicity island when compared with *P. jessenii* C2. It encompasses about 50,000 bp beginning at a gene encoding a sulfur carrier protein (KII37703) and ending with genes encoding Tn7 transposition proteins (KII37740-KII37743). This potential pathogenicity/fitness island harbored several copper resistance determinants including the *cus* determinant encoding CusABCRS (KII37706-37708, KII37711-37712) involved in periplasmic copper detoxification [32, 33]. In addition, genes encoding the P-type ATPase CopA, the multicopper oxidase CueO and CopBDG (KII37893, KII37715, KII37716, KII37709, KII37717) could be identified (Fig. 4). We also predicted specific GI for both *P. jessenii* H16 and *P. jessenii* C2 using the IsfindViewer [34]. Based on the automatic prediction algorithm two putative regions (coordinates KII37706-KII37717, KII37721-KII37737) were only identified in *P. jessenii* H16. Similar copper fitness islands could also be detected in *P.extremaustralis* 14-3b (AHIP00000000), isolated from a temporary pond in Antarctica; *Pseudomonas* *sp*.Ag1 (AKVH00000000) isolated from midguts of mosquitoes and *P. fluorescens* FH4 (AOHN00000000) [35–37]. This island also contained genes encoding the nickel efflux transporter NcrA (KII37721) and the transcriptional repressor NcrB (KII37723) [38]. Moreover, genes *merTRCAB* (KII37733-37737) encoding a mercury-resistance determinant are present on this island [39]. Many of the various putative GI contain functions related to mobility such as integrases or mobile genetic elements (MGE) which includes transposons and IS elements. As shown in *P. jessenii* H16, these putative GI have conferred this strain with additional heavy metal resistance capability, which may be transferred to other bacteria via Tn7 transposons and are highly relevant for adaption to this specific copper contaminated niche.

## Conclusion

The draft genome sequences of *P. jessenii* C2 isolated from low-Cu soil and *P. jessenii* H16 isolated from high-Cu soil were determined and described here. H16 provided an insight into the genomic basis of the observed higher copper resistance when compared with C2. Based on analysis and characterization of the genome, *P. jessenii* H16 is predicted to be resistant to a number of heavy metal(loid)s, such as Hg^2+^, Ni^2+^ Cr^2+^ and As^3+^. Comparative genomic analysis of those two strains suggested acquisition of a fitness island encoding numerous genes involved in conferring resistance to Cu and other metals as an important adaptive mechanism enabling survival of *P. jessenii* H16 in its Cu contaminated habitat. Possibly, *P. jessenii* H16 may have potential for bioremediation of copper contamination environments.

## Additional file

Additional file 1:
**Table S1.** Phenotypic characteristics of C2, H16 and phylogenetically related *P. jessenii* CIP 105275^T^. (DOCX 59 kb)

## Abbreviations

BGI: Beijing Genomics Institute
GI: Genomic island
MGE: Mobile genetic elements
