# Supplementary material for: Two draft genome sequences of Pseudomonas jessenii strains isolated from a copper contaminated site in Denmark
Source: Stand Genomic Sci. 2016 Nov 3;11:86. doi: 10.1186/s40793-016-0200-8 (PMC5100093; doi:10.1186/s40793-016-0200-8)
Supplement: Additional file 1: — Table S1. Phenotypic characteristics of C2, H16 and phylogenetically related P. jessenii CIP 105275T. (DOCX 59 kb) [file 40793_2016_200_MOESM1_ESM.docx]

**Supplementary Material**

**Table S1** Phenotypic characteristics of C2, H16 and phylogenetically related *P. jessenii* CIP 105275^T^

| **Characteristic** | ***P. jessenii* C2** | ***P. jessenii* H16** | ***P. jessenii* CIP 105275^T^** |
| --- | --- | --- | --- |
| Oxygen requirement | Aerobic | Aerobic | Aerobic^a^ |
| Gram stain | - | - | -^a^ |
| Salt requirement | 0-4% | 0-4% | 0-4%^a^ |
| **Production of** |  |  |  |
| Nitrate reductase | + | + | +^a^ |
| Urease | - | - | -^a^ |
| Indole | - | - | -^a^ |
| Lysine decarboxylase | - | - | -^a^ |
| Ornithine decarboxylase | + | + | -^a^ |
| Gelatinase | - | - | -^a^ |
| **Utilization of** |  |  |  |
| d-glucose | + | + | +^a^ |
| d-melibiose | + | + | +^a^ |
| d-sucrose | + | + | +^a^ |
| d-mannitol | + | + | +^a^ |
| sorbitol | - | - | +^a^ |
| l-rhamnose | + | - | +^a^ |
| inositol | + | + | +^a^ |
| trehalose | + | + | +^a^ |
| d-lyxose | + | + | +^a^ |
| l-arabinose | + | + | +^a^ |
| G + C content (mol%) | 58.87 | 59.02 | 58^a^ |
| Habitat | soil | Copper contaminated soil | mineral waters^a^ |

+: positive result, −: negative result.

^a^Data from Verhille *et al*.[22].

**GenBank Accession Summary**

| GenBank Accession | Summary |
| --- | --- |
| KII28988 | KII28988.1 is a bacterial sequences record containing linear AA (285 peptides) from Pseudomonas fluorescens strain H16. The record was created on January 22, 2015. It contains 4 features, including, 1 DNA coding regions. |
| KII37329 | KII37329.1 is a bacterial sequences record containing linear AA (148 peptides) from Pseudomonas fluorescens strain C2. The record was created on January 22, 2015. It contains 4 features, including, 1 DNA coding regions. |
| AOHN00000000 | AOHN00000000.1 is a bacterial sequences record containing linear, double-stranded DNA (64 bases) from Pseudomonas sp. strain FH4. The record was created on December 18, 2013 and last updated September 16, 2014. It contains 1 feature. |
| KII30277 | KII30277.1 is a bacterial sequences record containing linear AA (447 peptides) from Pseudomonas fluorescens strain H16. The record was created on January 22, 2015. It contains 6 features, including, 1 DNA coding regions. |
| KII37713 | KII37713.1 is a bacterial sequences record containing linear AA (178 peptides) from Pseudomonas fluorescens strain H16. The record was created on January 22, 2015. It contains 4 features, including, 1 DNA coding regions. |
| KII37736 | KII37736.1 is a bacterial sequences record containing linear AA (560 peptides) from Pseudomonas fluorescens strain H16. The record was created on January 22, 2015. It contains 8 features, including, 1 DNA coding regions. |
| KII28041 | KII28041.1 is a bacterial sequences record containing linear AA (276 peptides) from Pseudomonas fluorescens strain H16. The record was created on January 22, 2015. It contains 6 features, including, 1 DNA coding regions. |
| KII37706 | KII37706.1 is a bacterial sequences record containing linear AA (1,047 peptides) from Pseudomonas fluorescens strain H16. The record was created on January 22, 2015. It contains 4 features, including, 1 DNA coding regions. |
| KII29506 | KII29506.1 is a bacterial sequences record containing linear AA (315 peptides) from Pseudomonas fluorescens strain C2. The record was created on January 22, 2015. It contains 4 features, including, 1 DNA coding regions. |
| KII30013 | KII30013.1 is a bacterial sequences record containing linear AA (132 peptides) from Pseudomonas fluorescens strain C2. The record was created on January 22, 2015. It contains 8 features, including, 1 DNA coding regions. |
| PRJNA264019 | BioProject PRJNA264019: http://www.ncbi.nlm.nih.gov/bioproject/PRJNA264019 |
| KII32593 | KII32593.1 is a bacterial sequences record containing linear AA (65 peptides) from Pseudomonas fluorescens strain H16. The record was created on January 22, 2015. It contains 5 features, including, 1 DNA coding regions. |
| NC_012660 | NC_012660.1 is a contig record containing circular, double-stranded DNA (6,722,539 bases) from Pseudomonas fluorescens strain SBW25. The record was created on May 7, 2009 and last updated December 18, 2014. It contains 1 feature. |
| KII37723 | KII37723.1 is a bacterial sequences record containing linear AA (99 peptides) from Pseudomonas fluorescens strain H16. The record was created on January 22, 2015. It contains 9 features, including, 1 DNA coding regions. |
| KII37719 | KII37719.1 is a bacterial sequences record containing linear AA (321 peptides) from Pseudomonas fluorescens strain H16. The record was created on January 22, 2015. It contains 4 features, including, 1 DNA coding regions. |
| KII37717 | KII37717.1 is a bacterial sequences record containing linear AA (155 peptides) from Pseudomonas fluorescens strain H16. The record was created on January 22, 2015. It contains 4 features, including, 1 DNA coding regions. |
| KII37024 | KII37024.1 is a bacterial sequences record containing linear AA (816 peptides) from Pseudomonas fluorescens strain H16. The record was created on January 22, 2015. It contains 10 features, including, 1 DNA coding regions. |
| KII28043 | KII28043.1 is a bacterial sequences record containing linear AA (448 peptides) from Pseudomonas fluorescens strain H16. The record was created on January 22, 2015. It contains 13 features, including, 1 DNA coding regions. |
| KII36596 | KII36596.1 is a bacterial sequences record containing linear AA (230 peptides) from Pseudomonas fluorescens strain H16. The record was created on January 22, 2015. It contains 4 features, including, 1 DNA coding regions. |
| KII37740 | KII37740.1 is a bacterial sequences record containing linear AA (561 peptides) from Pseudomonas fluorescens strain H16. The record was created on January 22, 2015. It contains 4 features, including, 1 DNA coding regions. |
| KII37715 | KII37715.1 is a bacterial sequences record containing linear AA (642 peptides) from Pseudomonas fluorescens strain H16. The record was created on January 22, 2015. It contains 16 features, including, 1 DNA coding regions. |
| KII28679 | KII28679.1 is a bacterial sequences record containing linear AA (458 peptides) from Pseudomonas fluorescens strain H16. The record was created on January 22, 2015. It contains 10 features, including, 1 DNA coding regions. |
| KII37703 | KII37703.1 is a bacterial sequences record containing linear AA (594 peptides) from Pseudomonas fluorescens strain H16. The record was created on January 22, 2015. It contains 5 features, including, 1 DNA coding regions. |
| KII29565 | KII29565.1 is a bacterial sequences record containing linear AA (148 peptides) from Pseudomonas fluorescens strain H16. The record was created on January 22, 2015. It contains 4 features, including, 1 DNA coding regions. |
| JSAL00000000 | JSAL00000000.1 is a bacterial sequences record containing linear, double-stranded DNA (78 bases) from Pseudomonas fluorescens strain H16. The record was created on December 17, 2014 and last updated January 22, 2015. It contains 1 feature. |
| KII32595 | KII32595.1 is a bacterial sequences record containing linear AA (797 peptides) from Pseudomonas fluorescens strain H16. The record was created on January 22, 2015. It contains 11 features, including, 1 DNA coding regions. |
| KII36598 | KII36598.1 is a bacterial sequences record containing linear AA (115 peptides) from Pseudomonas fluorescens strain H16. The record was created on January 22, 2015. It contains 7 features, including, 1 DNA coding regions. |
| KII31669 | KII31669.1 is a bacterial sequences record containing linear AA (452 peptides) from Pseudomonas fluorescens strain C2. The record was created on January 22, 2015. It contains 5 features, including, 1 DNA coding regions. |
| KII37743 | KII37743.1 is a bacterial sequences record containing linear AA (285 peptides) from Pseudomonas fluorescens strain H16. The record was created on January 22, 2015. It contains 5 features, including, 1 DNA coding regions. |
| KII37716 | KII37716.1 is a bacterial sequences record containing linear AA (333 peptides) from Pseudomonas fluorescens strain H16. The record was created on January 22, 2015. It contains 5 features, including, 1 DNA coding regions. |
| KII37735 | KII37735.1 is a bacterial sequences record containing linear AA (144 peptides) from Pseudomonas fluorescens strain H16. The record was created on January 22, 2015. It contains 4 features, including, 1 DNA coding regions. |
| KII28042 | KII28042.1 is a bacterial sequences record containing linear AA (226 peptides) from Pseudomonas fluorescens strain H16. The record was created on January 22, 2015. It contains 11 features, including, 1 DNA coding regions. |
| KII31613 | KII31613.1 is a bacterial sequences record containing linear AA (285 peptides) from Pseudomonas fluorescens strain C2. The record was created on January 22, 2015. It contains 4 features, including, 1 DNA coding regions. |
| KII37707 | KII37707.1 is a bacterial sequences record containing linear AA (494 peptides) from Pseudomonas fluorescens strain H16. The record was created on January 22, 2015. It contains 6 features, including, 1 DNA coding regions. |
| KII29503 | KII29503.1 is a bacterial sequences record containing linear AA (281 peptides) from Pseudomonas fluorescens strain C2. The record was created on January 22, 2015. It contains 4 features, including, 1 DNA coding regions. |
| KII33434 | KII33434.1 is a bacterial sequences record containing linear AA (178 peptides) from Pseudomonas fluorescens strain C2. The record was created on January 22, 2015. It contains 5 features, including, 1 DNA coding regions. |
| KII37709 | KII37709.1 is a bacterial sequences record containing linear AA (312 peptides) from Pseudomonas fluorescens strain H16. The record was created on January 22, 2015. It contains 4 features, including, 1 DNA coding regions. |
| AF068259 | AF068259.1 is a bacterial sequences record containing linear, double-stranded DNA (1,515 bases) from Pseudomonas jessenii strain CIP 105274. The record was created on June 11, 1998 and last updated May 14, 2002. It contains 2 features, including, 1 rRNA features (16S ribosomal RNA). |
| KII37893 | KII37893.1 is a bacterial sequences record containing linear AA (676 peptides) from Pseudomonas fluorescens strain H16. The record was created on January 22, 2015. It contains 9 features, including, 1 DNA coding regions. |
| KII37733 | KII37733.1 is a bacterial sequences record containing linear AA (116 peptides) from Pseudomonas fluorescens strain H16. The record was created on January 22, 2015. It contains 4 features, including, 1 DNA coding regions. |
| AHIP00000000 | AHIP00000000.1 is a bacterial sequences record containing linear, double-stranded DNA (135 bases) from Pseudomonas extremaustralis substr. 14-3b strain 14-3. The record was created on January 10, 2012 and last updated April 11, 2014. It contains 1 feature. |
| KII36460 | KII36460.1 is a bacterial sequences record containing linear AA (427 peptides) from Pseudomonas fluorescens strain H16. The record was created on January 22, 2015. It contains 6 features, including, 1 DNA coding regions. |
| KII28987 | KII28987.1 is a bacterial sequences record containing linear AA (120 peptides) from Pseudomonas fluorescens strain H16. The record was created on January 22, 2015. It contains 4 features, including, 1 DNA coding regions. |
| JSAK00000000 | JSAK00000000.1 is a bacterial sequences record containing linear, double-stranded DNA (64 bases) from Pseudomonas fluorescens strain C2. The record was created on December 17, 2014 and last updated January 22, 2015. It contains 1 feature. |
| KII37737 | KII37737.1 is a bacterial sequences record containing linear AA (99 peptides) from Pseudomonas fluorescens strain H16. The record was created on January 22, 2015. It contains 4 features, including, 1 DNA coding regions. |
| KII35062 | KII35062.1 is a bacterial sequences record containing linear AA (769 peptides) from Pseudomonas fluorescens strain H16. The record was created on January 22, 2015. It contains 8 features, including, 1 DNA coding regions. |
| KII29505 | KII29505.1 is a bacterial sequences record containing linear AA (442 peptides) from Pseudomonas fluorescens strain C2. The record was created on January 22, 2015. It contains 8 features, including, 1 DNA coding regions. |
| KII30014 | KII30014.1 is a bacterial sequences record containing linear AA (797 peptides) from Pseudomonas fluorescens strain C2. The record was created on January 22, 2015. It contains 11 features, including, 1 DNA coding regions. |
| KII36597 | KII36597.1 is a bacterial sequences record containing linear AA (156 peptides) from Pseudomonas fluorescens strain H16. The record was created on January 22, 2015. It contains 5 features, including, 1 DNA coding regions. |
| KII37711 | KII37711.1 is a bacterial sequences record containing linear AA (231 peptides) from Pseudomonas fluorescens strain H16. The record was created on January 22, 2015. It contains 11 features, including, 1 DNA coding regions. |
| KII37721 | KII37721.1 is a bacterial sequences record containing linear AA (436 peptides) from Pseudomonas fluorescens strain H16. The record was created on January 22, 2015. It contains 6 features, including, 1 DNA coding regions. |
| KII37734 | KII37734.1 is a bacterial sequences record containing linear AA (91 peptides) from Pseudomonas fluorescens strain H16. The record was created on January 22, 2015. It contains 5 features, including, 1 DNA coding regions. |
| KII33436 | KII33436.1 is a bacterial sequences record containing linear AA (449 peptides) from Pseudomonas fluorescens strain C2. The record was created on January 22, 2015. It contains 13 features, including, 1 DNA coding regions. |
| KII29504 | KII29504.1 is a bacterial sequences record containing linear AA (543 peptides) from Pseudomonas fluorescens strain C2. The record was created on January 22, 2015. It contains 12 features, including 0 genes with 1 distinct annotations, 1 DNA coding regions. |
| KII28258 | KII28258.1 is a bacterial sequences record containing linear AA (458 peptides) from Pseudomonas fluorescens strain C2. The record was created on January 22, 2015. It contains 10 features, including, 1 DNA coding regions. |
| KII32596 | KII32596.1 is a bacterial sequences record containing linear AA (137 peptides) from Pseudomonas fluorescens strain H16. The record was created on January 22, 2015. It contains 8 features, including, 1 DNA coding regions. |
| KII34384 | KII34384.1 is a bacterial sequences record containing linear AA (753 peptides) from Pseudomonas fluorescens strain C2. The record was created on January 22, 2015. It contains 8 features, including, 1 DNA coding regions. |
| KII30016 | KII30016.1 is a bacterial sequences record containing linear AA (65 peptides) from Pseudomonas fluorescens strain C2. The record was created on January 22, 2015. It contains 5 features, including, 1 DNA coding regions. |
| KII37708 | KII37708.1 is a bacterial sequences record containing linear AA (418 peptides) from Pseudomonas fluorescens strain H16. The record was created on January 22, 2015. It contains 6 features, including, 1 DNA coding regions. |
| KII33435 | KII33435.1 is a bacterial sequences record containing linear AA (226 peptides) from Pseudomonas fluorescens strain C2. The record was created on January 22, 2015. It contains 11 features, including, 1 DNA coding regions. |
| KII37710 | KII37710.1 is a bacterial sequences record containing linear AA (462 peptides) from Pseudomonas fluorescens strain H16. The record was created on January 22, 2015. It contains 13 features, including, 1 DNA coding regions. |
| KII31612 | KII31612.1 is a bacterial sequences record containing linear AA (121 peptides) from Pseudomonas fluorescens strain C2. The record was created on January 22, 2015. It contains 4 features, including, 1 DNA coding regions. |

**Strain ID Summary**

| Strain ID | Summary |
| --- | --- |
| CIP 105275T | Collection Code: CIP  Collection Name: Pasteur Institute Collection, Biological Resource Center of Pasteur Institute (CRBIP)  Institution: CIP (Pasteur Institute Collection, Biological Resource Center of Pasteur Institute (CRBIP)) - France  Strain ID: CIP 105275T |

**Reference Search Summary**

| Name | Occurence |
| --- | --- |
